# Radiation Oncology and Medical Physics

**DOI:** 10.1155/2015/297158

**Published:** 2015-03-31

**Authors:** Tsair-Fwu Lee, Jack Yang, Cheng-Shie Wuu, An Liu, Fu-Min Fang, Shyh-An Yeh

**Affiliations:** ^1^Medical Physics & Informatics Laboratory of Electronics Engineering, National Kaohsiung University of Applied Sciences, Kaohsiung 80778, Taiwan; ^2^Graduate Institute of Clinical Medicine, Kaohsiung Medical University, Kaohsiung 807, Taiwan; ^3^Department of Radiation Oncology, Institute for Advanced Radiation Oncology, Monmouth Medical Center, Barnabas Health, Long Branch, NJ 07601, USA; ^4^Department of Radiation Oncology, Columbia University, New York, NY 10032, USA; ^5^Department of Radiation Oncology, City of Hope National Medical Center, Duarte, CA 91010, USA; ^6^Department of Radiation Oncology, Kaohsiung Chang Gung Memorial Hospital and Chang Gung University College of Medicine, Kaohsiung 83305, Taiwan; ^7^Department of Radiation Oncology of E-DA Hospital, Kaohsiung 82445, Taiwan; ^8^Department of Medical Imaging and Radiological Sciences of I-Shou University, Kaohsiung 82445, Taiwan

Radiation therapy has been evolving with technological advances in accelerator, computer, and imaging. As the planning target volumes (PTVs) are made increasingly conformal with the advent of three-dimensional conformal therapy (3D CRT) and intensity-modulated radiation therapy (IMRT), the requirements of precise PTVs localization and its dosimetric coverage for each treatment become more stringent. In 3D CRT, based on 3D anatomic information, treatment planning is designed to deliver dose distribution that conforms as closely as possible to the target volume and minimizes the dose to the critical organs. The advantage of IMRT is to treat a patient from a number of beams of different directions (or continuous arcs) with nonuniform fluences, which have been optimized to deliver a high dose to the target volume and an acceptable low dose to the surrounding normal tissue. The treatment planning program divides each beam into a large number of beamlets and determines their optimum fluences. This optimization process involves inverse treatment planning. Prediction of normal tissue complication probability (NTCP) after external beam radiotherapy (EBRT) is an important issue in the optimization of a treatment plan and should be considered because during EBRT a considerable volume of normal tissues receives radiation dose along with the tumor. Normal tissue complication probability (NTCP), in a normal tissue, is a function of delivered dose and irradiated volume of the normal tissue. Image-guided radiation therapy (IGRT) procedures employ imaging technology to help patient setup and target localization before and during treatment. Difficulties and problems associated with target localization may arise from inter- and intracranial variations in patient setup and anatomy, including shapes and volumes of treatment target and surrounding normal tissues. Some of current IGRT image guidance technologies include portal (MV) and radiographic (kV) imagers, in-room CT scanner, kV cone-beam CT, MV cone-beam CT, helical tomotherapy MVCT, and ultrasound.

Dose verification for complex treatment techniques such as IMRT, SRS/SBRT, and brachytherapy is crucial. These complex treatments present high dose gradient regions in the boundaries between the target and surrounding critical organs. Dose accuracy in these areas can be critical and may affect treatment outcome. A dose verification phantom designed for advanced technology in radiation therapy clinical trials can serve as a tool for quality assurance program.

Respiratory motion affects all tumor sites in the thorax and abdomen, although the disease of most prevalence and relevance for radiotherapy is lung cancer. Studies have shown that lung tumors can move several centimeters in any direction during irradiation. If respiratory motion is not accounted for, it causes artifacts during image acquisition. These artifacts cause distortion of the target volume and incorrect positional and volumetric information [1, 2]. A promising solution for obtaining high-quality CT data in the presence of respiratory motion is 4D CT or respiration-correlated CT (conventional and cone-beam approaches) [3]. The 4D images are reconstructed from scans acquired at each respiratory phase of the breathing cycle. Four-dimensional data can be analyzed to determine the mean tumor position, tumor range of motion for treatment planning [4], and the relation of tumor trajectory to other organs and to a respiration monitor. Respiratory gating involves the administration of radiation within a particular portion of the patient's breathing cycle, commonly referred to as the “gate.” The position and width of the gate within a respiratory cycle are determined by monitoring the patient's respiratory motion, using either an external respiration signal or internal fiducial markers. Since the beam is not continuously delivered, gated procedures are longer than nongated procedures.

Most radiation therapy treatment planning systems now incorporate three-dimensional anatomy information attained by computed tomography (CT) images for registration. These images can be fused with magnetic resonance imaging (MRI) or positron emission tomography (PET) images for better delineation of the target volume. MRI is considered superior to CT in soft-tissue discrimination such as central nervous system tumors and abnormalities in the brain. MRI is also used in imaging head and neck cancers, sarcomas, the prostate gland, and lymph nodes. On the other hand, CT imaging is more sensitive to bony structures and calcification.

Stereotactic radiosurgery (SRS) is a single-fraction radiation therapy procedure for treating intracranial lesions or a target volume close to critical structure, using a stereotactic apparatus and multiple small-field beams. The same procedure when used for delivering hypofractionated radiation treatment is called stereotactic radiotherapy (SRT). SRS and SRT involve three-dimensional imaging to localize the lesion and delivering a concentrated dose to the target volume while sparing as much as possible the normal tissues. Two common SRS techniques are available: linac-based X-ray knife and the Co-60 gamma-knife. Light ion (ion species with an atomic number less than or equal to 10) beams have also been used for SRS and SRT [5]. Local recurrence remains a problem in a relatively large number of patients after radiotherapy. One emerging method for dose escalation to improve local results is stereotactic body radiation therapy (SBRT). SBRT refers to a stereotactic radiotherapy procedure for treating extracranial tumors with a high doses per fraction (6 to 30 Gy), with a treatment regimen of five or fewer fractions [6–9]. SBRT procedures require meticulous planning, patient immobilization, organ motion management, and state-of-the-art image guidance techniques for target localization and geometric verification.

This special issue is focusing on the new development in cancer therapy, quality control, radiotherapy techniques, radiation dosimetry, and clinical outcome studies. This special issue included various topics which have been discussed from researchers of the following:

- clinical trials and outcome research;
- new technologies development and implementation;
- treatment delivery techniques;
- disease specific treatment discussion;
- radiation dosimetry analysis;
- radiation protection, shielding, and design;
- clinical therapy physics review and applications;
- molecular imaging application in radiation therapy;
- medical imaging;
- professional issues in medical, clinical, and biomedical physics;
- radiobiology;
- quality control and assurance;
- computing algorithm and optimization;
- quality of life analysis;
- radiation safety.

This editorial provides comprehensive introduction to technological advancements in radiation therapy and many important topics associated with these treatment technologies. Clinical data on these new technologies are provided by many institutions.

*Tsair-Fwu Lee* *Jack Yang* *Cheng-Shie Wuu* *An Liu* *Fu-Min Fang* *Shyh-An Yeh*
